# A systematic review of clinical guidelines on the management of acute, community-acquired CNS infections

**DOI:** 10.1186/s12916-019-1387-5

**Published:** 2019-09-06

**Authors:** Louise Sigfrid, Chelsea Perfect, Amanda Rojek, Kajsa-Stina Longuere, Sam Lipworth, Eli Harriss, James Lee, Alex Salam, Gail Carson, Herman Goossens, Peter Horby

**Affiliations:** 1 Centre for Tropical Medicine and Global Health, Nuffield Department of Medicine, University of Oxford, Oxford, UK; 2 University of North Carolina School of Medicine, Chapel Hill, NC USA; 3 UKERC, Imperial College London, London, UK; 4 Nuffield Department of Medicine, University of Oxford, Oxford, UK; 5 Bodleian Health Care Libraries, University of Oxford, Oxford, UK; 6 United Kingdom Public Health Rapid Support Team, London, UK; 7 University of Antwerp, Antwerp, Belgium

**Keywords:** CNS infections, Meningitis, Encephalitis, Meningoencephalitis, Guidelines, AGREE II appraisal

## Abstract

**Background:**

The epidemiology of CNS infections in Europe is dynamic, requiring that clinicians have access to up-to-date clinical management guidelines (CMGs) to aid identification of emerging infections and for improving quality and a degree of standardisation in diagnostic and clinical management practices. This paper presents a systematic review of CMGs for community-acquired CNS infections in Europe.

**Methods:**

A systematic review. Databases were searched from October 2004 to January 2019, supplemented by an electronic survey distributed to 115 clinicians in 33 European countries through the CLIN-Net clinical network of the COMBACTE-Net Innovative Medicines Initiative. Two reviewers screened records for inclusion, extracted data and assessed the quality using the AGREE II tool.

**Results:**

Twenty-six CMGs were identified, 14 addressing bacterial, ten viral and two both bacterial and viral CNS infections. Ten CMGs were rated high quality, 12 medium and four low. Variations were identified in the definition of clinical case definitions, risk groups, recommendations for differential diagnostics and antimicrobial therapy, particularly for paediatric and elderly populations.

**Conclusion:**

We identified variations in the quality and recommendations of CMGs for community-acquired CNS infections in use across Europe. A harmonised European “framework-CMG” with adaptation to local epidemiology and risks may improve access to up-to-date CMGs and the early identification and management of (re-)emerging CNS infections with epidemic potential.

**Electronic supplementary material:**

The online version of this article (10.1186/s12916-019-1387-5) contains supplementary material, which is available to authorized users.

## Introduction

Endemic, epidemic and emerging infectious diseases, including antimicrobial resistant organisms, remain a serious, cross-border threat to health in Europe. The response to these threats needs to be evidence-based and coordinated, and whilst Europe-wide efforts have been made to link and harmonise public health responses, much less has been done in the clinical sphere. The EU-funded Platform for European Preparedness Against R(e-)emerging Epidemics (PREPARE) was established to promote harmonised clinical research studies on infectious diseases with epidemic potential in order to improve patient outcomes and inform public health responses. One issue identified by PREPARE was the lack of understanding of variations in clinical practice across Europe, which may hamper the interpretation of clinical and surveillance data on emerging infectious threats with epidemic potential and impede the implementation of cross-border clinical research.

Central nervous system (CNS) infections continue to affect populations worldwide with high morbidity, mortality and risk of long-term sequelae and are also associated with a range of emerging and re-emerging viral threats to Europe, such as West Nile virus, Toscana virus, measles and enteroviruses [1, 2]. The epidemiology of community-acquired CNS infections is neither fixed nor homogeneous, with changes over time and between locations. The introduction of vaccines has reduced the burden of the two most common etiological agents for bacterial meningitis in adults and older children, *Streptococcus pneumoniae* and *Neisseria meningitidis* [3, 4]. *Haemophilus influenzae* type B (Hib) is also becoming a rare cause of meningitis in Europe [5]. However, reports of serotype replacement and an increased rate of reduced sensitivity to antimicrobial agents of *S. pneumoniae,* with varying rates across the region, are a cause of concern, which requires antibiotic regimes to be tailored to regions and travel [3, 5]. Neonatal meningitis is associated with high morbidity and higher incidence compared to older age groups [6]. In neonates, common pathophysiology are primary bloodstream infections with secondary haematogenous distribution to the CNS [6] most commonly caused by *Streptococcus agalactiae* (group B streptococcus; GBS) or *Escherichia coli* [3]. Encephalitis, an inflammation of the brain parenchyma associated with high morbidity and risk of long-term sequelae, is commonly caused by viruses [7]. It is estimated that 40 to 60% of cases remain without an aetiological diagnosis [8, 9]. This may partly be due to a lack of consensus on clinical case definitions and standardised diagnostic approaches [10]. The most commonly diagnosed causes of viral CNS infections in Europe are *Herpes simplex virus* (HSVs), enteroviruses, *Varicella-zoster virus* (VZV) and arthropod-borne viruses (arboviruses) [11]. The epidemiology of encephalitis is constantly evolving [11], and emerging infectious diseases may present as undifferentiated CNS infections [12]. This is ilustrated by the re-emergence of *West Nile virus* (WNV) in south-eastern Europe and the emergence of *Toscana virus* as a leading cause of aseptic meningitis in regions in southern Europe during the summer [13, 14]. Another cause of concern are recent outbreaks of enterovirus-associated severe neurological disease which cause a strain on paediatric intensive care units [15].

Clinical case definitions and clinical management guidelines (CMGs) are important tools for identifying emerging infectious diseases, informing diagnostic and clinical management and providing a degree of standardisation in clinical management practices. In addition, harmonisation of diagnostic and clinical management practices can inform public health outbreak responses and facilitate the design and interpretation of multi-country research, which is a necessity for adequately powered studies of comparatively rare diseases such as CNS infections.

The aim of this review is to identify variations in practices which might be a barrier to the early identification and characterisation of emerging CNS infections with epidemic potential and the implementation of cross-border clinical research as well as public health responses. This is, to our knowledge, the first systematic review and quality appraisal of European CMGs for viral and bacterial CNS infections.

## Methods

The systematic review was completed based on a protocol registered in the PROSPERO International prospective register of systematic reviews (ID: CRD42014014212). The protocol was informed by infectious disease specialists and systematic reviewers.

### Search strategy

One reviewer conducted the first electronic database search (PubMed, National Guideline Learning Centre, International Guideline Library, TRIP Database) from October 2004 to October 2014. Search terms were as follows: (central nervous system infection [MeSH Terms]) AND (clinical guideline OR clinical practice guideline OR physician guideline OR bedside clinical guideline OR clinical management guideline OR clinical practice protocol OR physician protocol OR clinical management protocol) AND (“last 10 years” [PDat]). An information specialist performed a second updated electronic search of Ovid MEDLINE, Ovid Embase, PubMed, TRIP Database and Google using the exploded thesaurus term “exp Central Nervous System Infections/”, and the free text terms meningitis, encephalitis, meningoencephalitis, combined with a search filter for guidelines to 22 June 2017.

Search terms:

(“Central Nervous System Infections”[Mesh]) OR meningitis [Title/Abstract]) OR meningoencephalitis [Title/Abstract]) OR encephalitis [Title/Abstract])) AND (guideline [Title]) OR guidelines [Title]) OR guidance [Title]) OR protocol [Title]) OR protocols [Title]) OR ((“Guideline” [Publication Type] OR “Guidelines as Topic”[Mesh]) OR “Practice Guideline” [Publication Type])).

TRIP Database and Google were also searched for “meningitis guideline*”, “encephalitis guideline*” and “meningoencephalitis guideline*” up to 31 January 2019. The electronic database searches were supplemented by searching the references of included CMGs and CMGs identified through a brief electronic survey which was e-mailed to 115 clinicians in 33 European countries, through the CLIN-Net clinical network of the COMBACTE-Net Innovative Medicines Initiative [16, 17]. The survey asked clinicians which CMGs they used in their daily practice to identify and manage patients presenting with syndromes of acute, community-acquired CNS infections, and asked them to submit the CMGs via hyperlink or by e-mail. The survey was open from 20 June to 30 December 2016, with two electronic reminders.

### Eligibility criteria

Two reviewers screened the title, abstract and full-text guidelines for inclusion. CMGs covering diagnostics and/or clinical management of suspected community-acquired bacterial or viral CNS infections which were aimed at or used by clinicians in Europe and published from 2004 onwards were included. The CMG produced by Médicins Sans Frontières (MSF) aimed at field settings globally was included, as it could be used in Europe in emergency situations. There were no language limitations. Guidelines published in non-English languages were translated using Google Translate and reviewed by a reviewer with good to excellent knowledge of the language. Guidelines that were not aimed at European populations were excluded, unless a clinician responding to the survey reported using them. General antibiotic and local standard operating policies were excluded. Guidelines focused only on patients with specific risk factors, such as HIV, were excluded.

### Data extraction

A standardised form for data extraction covering case definitions, diagnostic methods, differential diagnostics and medical management recommendations was developed. One reviewer extracted data from the CMGs and a second reviewer checked the data.

### Quality appraisal

The CMGs were critically appraised by two reviewers independently using the Appraisal of Guidelines for Research and Evaluation II (AGREE II) Instrument [18, 19]. The quality was assessed independently by each reviewer for six domains: (1) scope and purpose, (2) stakeholder involvement, (3) rigour of development, (4) clarity of presentation, (5) applicability and (6) editorial independence, and through an overall quality score. Efforts were made to find additional information online on associated webpages for CMGs with limited information about the methodology used. Within each domain, there were a number of sub-criteria to score from 1 to 7 (Additional file 1). A score of one was assigned if there was no information or the criteria was not met; a score of seven when the criteria were met. These scores were summarised for each domain, and the total score for the domain calculated as the percentage of the total possible score for that domain. The final score for each domain was calculated as the average of the reviewers’ scores. Each CMG was also given a total overall quality assessment score based on the average score for all the domains (7 being the highest quality) together with a recommendation for use with or without further modifications.

## Results

### Clinical management guidelines

**Fig. 1:**
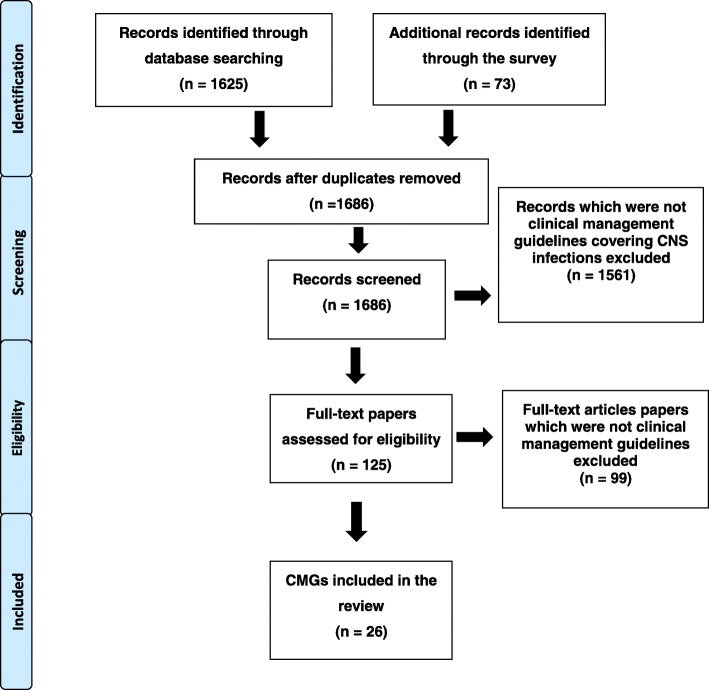
PRISMA flowchart

**Fig. 2:**
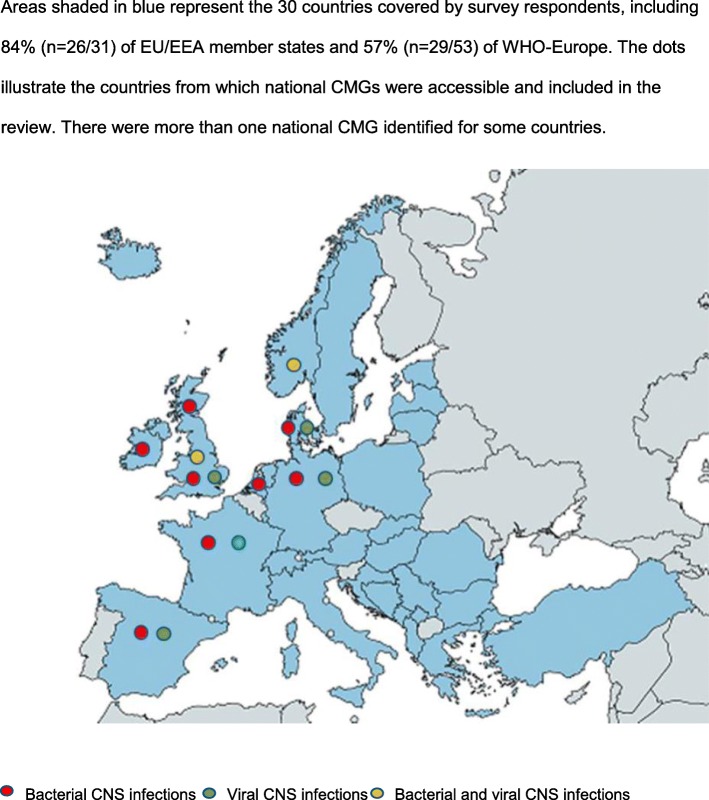
Survey response and national CMG coverage

A total of 26 CMGs covering community-acquired suspected bacterial or viral CNS infections met the inclusion criteria for the review (Fig. 1). The 26 CMGs were produced in Denmark (*n* = 2), France (*n* = 2), Germany (*n* = 2), Ireland (*n* = 1), the Netherlands (*n* = 1), Norway (*n* = 1), Scotland (*n* = 1), Spain (*n* = 3), the UK (*n* = 6), Europe (*n* = 3), the USA (*n* = 3) and MSF (*n* = 1) (Fig. 2). Ten focused on viral encephalitis/meningoencephalitis, 14 on bacterial meningitis and two on both (Additional file 2).

Of the 76 clinicians (*n* = 76/115, 66%) from 30 European countries who responded to the survey, 29% reported using CMGs produced by US-based, 27% by national, 23% by local and 14% by European organisations. There were no national CMGs identified from those countries where respondents reported using international CMGs.

### Quality appraisal

The overall quality of the CMGs ranged from three to seven (Table 1). Ten CMGs were assessed as of high quality (scores 6–7), 12 of medium (scores 4–5) and four of low quality (score ≤ 3). Six CMGs which focused on bacterial CNS infections gained the maximum quality score. Three CMGs scored below 4 and were assessed as in need of modifications in order to adhere to the AGREE II guideline development standards [19]. These modifications included additional information such as the methodology used to identify evidence and formulate recommendations, explicit links between evidence and recommendations and information about stakeholder engagement and peer review. Wide variations in scores between CMGs were seen for “rigour of development”. Seven CMGs scored above 75% for this domain, six of which covered bacterial [2, 5, 20–24], and one viral [22] CNS infections. Most CMGs scored well on “clarity of presentation” and “scope and purpose”. Some score variations may be due to a lack of information presented, such as on stakeholder engagement, editorial independence and plans for regular revisions. This may partly explain the general low scores for “applicability” and “editorial independence”.

**Table 1:** AGREE II scores

Guidelines	Scope and purpose (%)	Stakeholder involvement (%)	Rigour of development (%)	Clarity of presentation (%)	Applicability (%)	Editorial independence (%)	Overall quality
**Viral aetiology**							
IEC	91.7	63.9	40.6	91.7	52.1	50.0	5
EFNS	83.3	52.8	55.2	91.7	47.9	25.0	5
DNS	38.7	27.7	22.1	41.8	16.6	0.0	3
DGN: VM	69.4	44.4	50.0	75.0	20.8	66.7	5
IDSA	72.2	50.0	58.3	86.1	18.8	83.3	6
PHE: VE	88.9	75.0	44.8	86.1	31.3	50.0	6
BIA: ABN	100	83.3	94.8	94.4	47.9	66.7	6
BIA: ABN/BPAIIG	100	83.3	74.0	91.7	54.2	0.0	6
AEPED	61.1	33.3	24.0	55.6	20.8	8.3	3
SPILF	78	41.5	45.5	75.0	4.0	8.3	4
Median	80.7	51.4	47.8	86.1	26.1	37.5	5.0
Range	(39–100)	(22–83)	(22–95)	(42–94)	(17–54)	(0–83)	(3–6)
**Viral and bacterial aetiology**							
NNF	75.0	52.8	37.5	88.9	6.3	29.2	4
PHE: ME	97.2	69.4	28.1	94.4	43.8	83.3	5
Median	86.1	61.1	32.8	91.7	25.1	56.3	4.5
Range	(75–97)	(52–69)	(28–38)	(89–94)	(6–44)	(29–83)	(4–5)
**Bacterial aetiology**							
EFNS	94.4	52.8	47.9	97.2	43.8	0.0	5
ESCMID	100	83.3	88.5	88.9	47.9	66.7	7
DSI	75.0	36.1	20.8	86.1	45.8	0.0	4
SPILF	83.3	38.9	44.8	88.9	25.0	0.0	4
DGN: BM	69.4	44.4	50.0	75.0	20.8	66.7	5
HPSC	100	66.7	45.8	94.4	39.6	0.0	5
NVN	97.2	94.4	87.6	97.2	66.7	29.2	7
MHSSE	100	94.4	90.6	86.1	43.8	50.0	7
NICE	100	86.1	95.8	88.9	79.2	70.8	7
UKJSS	100	88.9	77.1	94.4	47.9	50.0	7
SIGN	91.7	91.7	79.2	86.1	37.5	45.8	7
IDSA	75.0	50.0	59.4	86.1	10.4	54.2	5
AEPED	61.1	33.3	19.8	83.3	20.8	0.0	3
MSF	88.9	47.2	9.4	91.7	50.0	0.0	4
Median	93.1	59.8	54.7	88.9	43.8	37.5	5
Range	(61–100)	(33–94)	(9–96)	(75–97)	(10–79)	(0–71)	(3–7)

*Abbreviations*: *IEC* International Encephalitis Consortium, *EFNS* European Federation of Neurological Societies, *DNS* Dansk Neurologisk Selskab, *DGN: VM* Deutsche Gesellschaft für Neurologie: Virale Meningoeczephalitis, *PHE: VE* Public Health England: Viral Encephalitis, *BIA: ABN* British Infection Association: Association of British Neurologists, *BPAIIG* The British Paediatric Allergy Immunology and Infections Group, *AEPED* Asociación Española de Pediatría, *SPILF* Société de Pathologie Infectieuse de Langue Française, *PHE: ME* Public Health England: Meningoencephalitis, *NNF* Norsk Nevrologisk Forening, *ESCMID* European Society of Clinical Microbiology and Infectious Diseases, *DSI* Dansk Selskab for Infektionsmedicin, *DGN: BM* Deutsche Gesellschaft für Neurologie: Bakterielle Meningoenzephalitis, *HPSC* Health Protection Surveillance Centre, *NVN* Nederlandse Vereniging voor Neurologie, *MHSSE* Ministry of Health, Social Services and quality, *NICE* The National Institute for Health and Care Excellence, *UKJSS* UK Joint Specialist Societies, *SIGN* Scottish Intercollegiate Guidelines Network, *IDSA* Infectious Diseases Society of America, *MSF* Médecins Sans Frontières

### Signs and symptoms at presentation

#### Viral encephalitis/meningoencephalitis

Encephalitis is an inflammation of the brain parenchyma associated with neurological dysfunction [7, 10], which is reflected in the syndromic presentation. Meningoencephalitis affects both the brain parenchyma and meninges [25]. Four of the 12 CMGs covering viral aetiologies described symptoms at presentation in adults [7, 10, 22, 26], four in paediatric populations [7, 10, 27, 28] and six in unspecified populations [25, 29–33] (Table 2). Most of the guidelines cited focal neurological signs, seizures, fever, altered levels of consciousness (ALOC) and changes to personality or behaviour as signs and symptoms of encephalitis in both children and adults. It was noted that objective fever might be lacking at the time of assessment [10, 26] particularly in immunosuppressed patients [10].

**Table 2:** Common signs and symptoms at presentation. The table shows the proportion of CMGs that each specific signs or symptoms was described in

**Bacterial CNS infections**					
Neonates/infants (≤ 1 year) (*n* = 8 CMGs)		Children (< 16–18 years) (*n* = 10 CMGs)		Adults and unspecified age (*n* = 10 CMGs)	
Signs and symptoms	*n* (%)	Signs and symptoms	*n* (%)	Signs and symptoms	*n* (%)
Irritability	8 (100)	Neck stiffness	10 (100)	Fever	10 (100)
Fever	8 (100)	Headache	9 (90)	Headache	10 (100)
Poor appetite/feeding	8 (100)	Fever	9 (90)	Neck stiffness	10 (100)
Bulging fontanelle	6 (75)	Petechial rash/purpura	8 (80)	AMS/ALOC	6 (60)
Petechial rash/purpura	6 (75)	AMS	7 (70)	Petechial rash/purpura	5 (50)
Lethargy	6 (75)	Nausea/vomiting	6 (60)	Focal neurological signs	5 (50)
Nausea/vomiting	5 (63)	Photophobia	6 (60)	Seizures	4 (40)
Neck stiffness	5 (63)	Kernig’s sign	5 (50)	Photophobia	3 (30)
Seizures	4 (50)	Brudzinski’s sign	5 (50)	Nausea/vomiting	3 (30)
Apnoea or respiratory distress	4 (50)	Cold hands/feet	4 (40)		
Hypothermia	4 (50)	Abnormal skin colour	4 (40)		
Confusion	3 (38)	Focal neurological signs	3 (30)		
Photophobia	3 (38)	Poor appetite	3 (30)		
Abnormal skin colour	3 (38)	Leg pain	2 (20)		
Kernig’s sign	2 (25)	Lethargy	2 (20)		
Brudzinski’s sign	2 (25)	Irritability	2 (20)		
**Viral CNS infections**					
Children (*n* = 4 CMGs)		Adults and unspecified age (*n* = 10 CMGs)			
Signs and symptoms	*n* (%)	Signs and symptoms		*n* (%)	
Seizures	4 (100)	Seizures		10 (100)	
Focal neurological signs	4 (100)	Focal neurological signs		10 (100)	
Fever	4 (100)	Fever		9 (90)	
ALOC	4 (100)	ALOC		9 (90)	
Changes in personality or behaviour	4 (100)	Changes in personality or behaviour		8 (80)	
Headache	1 (25)	Rash		5 (50)	
		Headache		4 (40)	
		Vomiting		1 (10)	
		Memory changes		1 (10)	

*Abbreviations: AL*OC altered level of consciousness, *AMS* altered mental status

#### Bacterial meningitis

Nine CMGs covering bacterial CNS meningitis presented symptoms at presentation in adults [2, 5, 23, 34–39], ten in children [5, 20, 21, 23, 24, 34–36, 38, 40], eight in infants [5, 20, 23, 24, 34, 35, 38, 40] and one in unspecified populations [31] (Table 2). The classic symptoms of fever, headache, neck stiffness, followed by altered mental status and petechial rash, were most frequently described for adults and older children. However, for children, there were a wider range of symptoms reported, with some such as leg pain and cold hands and feet specifically described in children. The symptoms in infants and neonates were more general and can be undistinguishable from sepsis [20]. Irritability, fever and poor feeding were most commonly described, followed by bulging fontanelle, petechial rash and lethargy. It was also highlighted by several CMGs that although high fever can be a sign of severity, fever might not always be present [24, 34], particularly in neonates [5, 23, 24, 40]. This is consistent with the conclusions in the recent CMG by the European Society for Microbiology and Infectious Diseases (ESCMID) that, based on evidence reviews, there are no clinical signs and symptoms that are present in all children [5]. It was also highlighted that the classic signs of meningitis are not always present in adults either [23]. Therefore, bacterial meningitis cannot be ruled out based on the absence of classical signs and symptoms alone [5].

### Diagnostic methods

#### Viral encephalitis/meningoencephalitis

Encephalitis is generally diagnosed based on a combination of clinical, laboratory and neuroimaging features [10, 22]. Cerebrospinal fluid (CSF) investigation with polymerase chain reaction (PCR) can differentiate common aetiologies [41–43]. Epidemiological factors can guide further investigations [22]. Most CMGs recommended urgent blood and CSF sampling, unless lumbar puncture (LP) is contraindicated due to signs of raised intracranial pressure (ICP) (Table 3). Magnetic resonance imaging (MRI) was also recommended when possible, or alternatively computed tomography (CT), since MRI can be useful to detect early changes and for excluding alternative causes and is more sensitive and specific compared to CT [10]. It was highlighted that although some specific changes on MRI have been associated with certain aetiological agents (e.g. HSV and arboviruses), it does not always assist in differentiation and findings might initially be normal [7]. EEG to aid diagnostics was mainly recommended for patients displaying certain symptoms such as altered behaviour or non-convulsive seizures, to exclude non-infectious causes [22].

**Table 3:**
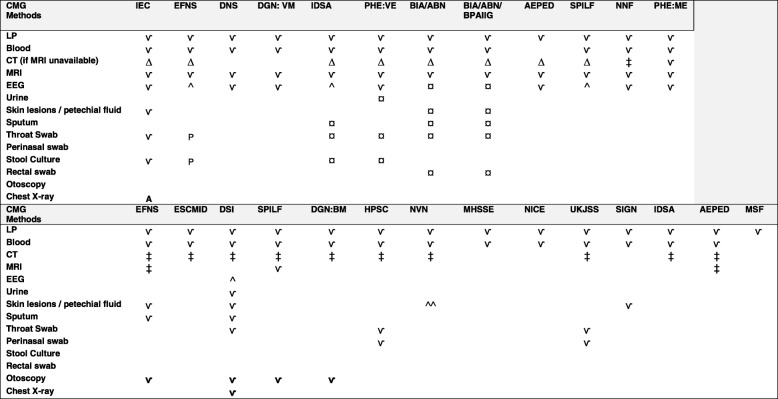
Diagnostic methods

The table shows the diagnostic methods recommended for patients with suspected viral or bacterial aetiologies. CT should not delay antibiotic treatment for suspected bacterial meningitis

*Abbreviations*: *IEC* International Encephalitis Consortium, *EFNS* European Federation of Neurological Societies, *DNS* Dansk Neurologisk Selskab, *DGN: VM* Deutsche Gesellschaft für Neurologie: Virale Meningoeczephalitis, *PHE: VE* Public Health England: Viral Encephalitis, *BIA: ABN* British Infection Association: Association of British Neurologists, *BPAIIG* The British Paediatric Allergy Immunology and Infections Group, *AEPED* Asociación Española de Pediatría, *SPILF* Société de Pathologie Infectieuse de Langue Française, *PHE: ME* Public Health England: Meningoencephalitis, *NNF*: Norsk Nevrologisk Forening, *ESCMID* European Society of Clinical Microbiology and Infectious Diseases, *DSI* Dansk Selskab for Infektionsmedicin, *DGN: BM* Deutsche Gesellschaft für Neurologie: Bakterielle Meningoenzephalitis, *HPSC* Health Protection Surveillance Centre, *NVN* Nederlandse Vereniging voor Neurologie, *MHSSE* Ministry of Health, Social Services and Equality, *NICE* The National Institute for Health and Care Excellence, *UKJSS* UK Joint Specialist Societies, *SIGN* Scottish Intercollegiate Guidelines Network, *IDSA* Infectious Diseases Society of America, *MSF* Médecins Sans Frontières, *CT* computerised tomography, *MRI* magnetic resonance imaging, *EEG* electroencephalogram

*Adults only

**Children only

^∆^If MRI unavailable

^¤^Depending on clinical and epidemiological features

^‡^Prior to lumbar puncture only if signs of elevated intracranial pressure (ICP)

^^^If seizures or consciousness disorder

^^^^If LP contraindicated

*Viral encephalitis/meningoencephalitis, Bacterial meningitis*

#### Bacterial meningitis

All CMGs covering bacterial CNS infections recommended urgent LP and most recommended blood sampling (Table 3), since a positive CSF culture is confirmative of bacterial meningitis and enables in vitro testing of antimicrobial susceptibility to optimise antibiotic treatment, and urgent LP increases diagnostic chances [5]. If CSF examination is not possible, serum markers of inflammation and blood cultures, especially if taken prior to antibiotics, can support the diagnosis and immunochromatographic antigen testing and PCR can provide additional information [5]. The CMG by MSF did not recommend LP for new cases in an epidemic context when a meningococcal aetiology was confirmed [38]. Sixty-nine percent (*n* = 11/16) recommended a CT scan before LP if clinical signs of raised ICP [2, 5, 23, 31, 34–37, 39, 40, 44]. In contrast, the CMG by NICE stated that CT is unreliable for identifying raised ICP and recommended clinical assessment instead of CT [24].

### Differential diagnostics

#### Viral encephalitis/meningoencephalitis

There were wide variations in the differential diagnostic recommendations for suspected viral CNS infections, reflecting the many potential causative agents (Table 4). All CMGs recommended testing for HSV and VZV and most also for enteroviruses and human immunodeficiency virus (HIV). Fifty percent recommended testing for *parechoviruses* [10, 22, 25, 28, 30, 31], with one CMG specifying only in children [31] and three only in children under 3 years old [10, 25, 30]. Other CMGs recommended testing for *Epstein-Barr virus* (EBV), *human herpesvirus* [6, 7], adenoviruses and, depending on season or exposure, arboviruses. Testing for influenza, mumps, measles and rubella were also recommended, especially during an on-going epidemic [30]. Many, but not all CMGs provided differential diagnostic tables based on risk factors, such as age, immune status, travel, animal contact and seasonality [7, 10, 22, 25, 27–29]. It was noted by some that PCR, e.g. for HSV, the most commonly diagnosed aetiological agent, can be falsely negative, especially in children and early disease course [10]. If a test is negative and still concerns about the diagnosis, it was recommended to take a second CSF sample within 3 to 7 days [7, 10, 22]; one CMG specified a minimum of 4 days after onset of neurological symptoms [26].

**Table 4:** Diagnostics recommendations for suspected viral encephalitis/meningoencephalitis

CMG	Differential diagnostic recommendations
IEC*	Children: HSV-1, HSV-2, enteroviruses, EBV, parechovirus (< 3 years), *Mycoplasma pneumoniae,* TBEV, WNV (S. Europe), TOSV (S. Europe) Adults: HSV-1, HSV-2, enteroviruses, VZV*, Treponema pallidum*, *Cryptococcus*, HIV, TBEV, WNV (S. Europe), TOSV (S. Europe) Others based on epidemiological and clinical features.
EFNS^^^	Unspecified: HSV-1, HSV-2, VZV, HHV 6/7, CMV, EBV, JCV, Dengue virus, respiratory viruses, RSV, HIV, adenovirus, influenza A and B, rotavirus*, Coxsackie B5*, non-typed enterovirus, *Parainfluenza 1* virus
DNS	Unspecified: HSV, VZV, HIV, EBV/CMV, syphilis, HHV 6–7, toxoplasma
DGN: VM	Unspecified: HSV, VZV, CMV, coxsackievirus, echovirus, adenovirus, phlebovirus, measles, TBE, mumps, EBV, rubella, enterovirus 71, HIV, parvovirus B19, HHV-6, Dengue virus, FSME, rabies, vaccinia, Lassa virus, Japanese encephalitis, WNV, polioviruses, hantavirus, filovirus Others based on epidemiological and clinical features.
IDSA*	Neonates: HSV 2, CMV, rubella virus, *L. monocytogenes, T. pallidum, Toxoplasma gondii* Infants and Children: *Eastern equine encephalitis virus, Japanese encephalitis virus, Murray* *Valley encephalitis virus*, influenza virus, *La Crosse virus* Unspecified: HSV, WNV, TBEV, *A. phagocytophilum, B.burgdorferi* Elderly: *Eastern equine encephalitis virus, St. Louis encephalitis virus,* WNV, sporadic Creutzfeldt-Jakob Disease, *L. monocytogenes* Person-to-person transmission: VZV, *Venezuelan equine encephalitis virus* (rare), poliovirus, non-polio enteroviruses, measles virus*,* mumps virus, rubella virus, EBV, HHV 6, B virus, WNV (transfusion transplantation, breast feeding), HIV, *Nipah virus*, rabies virus (transplantation), influenza virus, *M. pneumoniae, Mycobacterium tuberculosis, T. pallidum.* Others based on specific clinical findings, epidemiology and risk factors.
PHE: VE*	Children and adults: HSV-1, HSV-2, VZV, enteroviruses, parechovirus (< 3 years), HHV-6 (< 3 years), mumps (if epidemic), Influenza A, B (if epidemic), HIV, Others based on specific epidemiology and risk factors
BIA/ABN	Adults: HSV-1, HSV-2, VZV, enteroviruses, parechovirus, measles virus, mumps virus, TBEV (eastern Europe), WNV (southern Europe), HIV, EBV (immunocompromised), CMV (immunocompromised) influenza virus (rare), adenovirus (rare), *Erythrovirus B19* (rare), lymphocytic choreomeningitis virus (rare), rubella virus (rare) Others based on epidemiological and clinical features.
BIA/ABN/BPAIIG	Children: HSV-1, HSV-2, VZV, enteroviruses, parechovirus*,* HHV 6/7, measles virus, mumps virus, rotavirus, HIV, TBEV (eastern Europe), WNV (southern Europe), HIV, EBV (immunocompromised), CMV (immunocompromised) influenza virus (rare), adenovirus (rare), *Erythrovirus B19* (rare), lymphocytic choreomeningitis virus (rare), rubella virus (rare) Others based on epidemiological and clinical features.
AEPED*	Congenital: *Lymphocytic Choriomeningitis virus,* CMV, measles virus, *T. gondii, T. pallidum,* Neonates (< 1 month): HSV, enteroviruses, adenovirus, *Citrobacter* spp., GBS, *L. monocytogenes* Infants and children > 1 month: HSV, enteroviruses, arboviruses, EBV, adenovirus, HIV, *M. pneumoniae*, *Borrelia burgdorferi*, *Bartonella henselae*, *Rickettsia rickettsii.* Others based on epidemiological and clinical features.
SPILF*^^^	Adults (metropolitan France): HSV, VZV, HIV, enterovirus, *L. monocytogenes (higher risk > 65 years)*, *M. tuberculosis (higher risk > 75 years)*
NNF*	Unspecified: HSV-1, HSV-2, VZV, EBV, HHV-6, enteroviruses*,* parechovirus (children), adenovirus, *Influenza A*, measles, mumps, rubella, HIV, *Hepatitis E*, *Japanese B*, WNV, rabies virus, TBE, *Eastern equine encephalitis*, *S. pneumoniae*, *B. burgdorferi*, *M. pneumoniae*, *Chlamydia trachomatis*, *L. monocytogenes*, *M. tuberculosis*, cerebral malaria, Cystericosis. Others based on immune status.
PHE: ME*	Unspecified: HIV, HSV-1, HSV-2, VZV, enteroviruses, parechovirus (< 3 years), *N. meningitidis*, *S. pneumoniae*, *H. influenzae*, *E. coli, L. monocytogenes*, GBS

*Abbreviations*: *HSV* Herpes simplex virus, *VZV* Varicella-zoster virus, *HIV* human immunodeficiency virus, *HHV* human herpesvirus, *CMV* Cytomegalovirus, *EBV* Epstein-Barr virus, *RSV* respiratory syncytial virus, *TBEV* tick-borne encephalitis virus, *WNV* West Nile virus, *JEV* Japanese encephalitis virus, *IEC* International Encephalitis Consortium, *EFNS* European Federation of Neurological Societies, *DNS* Dansk Neurologisk Selskab, *DGN: VM* Deutsche Gesellschaft für Neurologie: Virale Meningoeczephalitis, *PHE: VE* Public Health England:Viral Encephalitis, *BIA: ABN* British Infection Association: Association of British Neurologists, *BPAIIG* The British Paediatric Allergy Immunology and Infections Group, *AEPED* Asociación Española de Pediatría, *SPILF* Société de Pathologie Infectieuse de Langue Française, *NNF* Norsk Nevrologisk Forening, *PHE: ME* Public Health England: Meningoencephalitis

*Includes non-viral differential diagnostic recommendations

^^^Differential diagnostic recommendations not clearly defined

#### Bacterial meningitis

There was consensus on the initial differential diagnostics for adults and older children presenting with syndromes suggestive of bacterial meningitis (Table 5). Besides two CMGs which only focused on *N. meningitidis* [20, 21], all recommended testing for *N. meningitidis* and *S. pneumoniae* in adults and children, but with variations in recommendations for Hib testing. Most also recommended testing for *L. monocytogenes* in adults; four specified for adults over 50 years [5, 31, 39, 44], and one in adults over 60 years [2]. Additional risk groups for *L. monocytogenes* were specified as adults over 18 years old with chronic conditions causing immunosuppression [2, 5, 25, 36, 39].

**Table 5:** Differential diagnostic recommendations for suspected bacterial meningitis

Pathogen	Age group	EFNS Europe	ESCMID Europe	DSI Denmark	SPILF France	DGN: BM^^^ Germany	HPSC Ireland	NVN Netherlands	NICE UK	UKJSS^^^^ UK	IDSA USA/Global	AEPED Spain	MSF Global	NNF^^^^	PHE: ME
*S. pneumoniae*	Neonates		(very rare)				N		N				> 7 days		NS
	Paediatric	P	P		P	P	NS	1–12 months	≥ 3 months		P	P	> 3 months	> 2 years	NS
	Adults	A	A	A	A	A	NS	A		A	A		A	A	NS
*N. meningitidis*	Neonates						N								NS
	Paediatric	P	P		P	P	NS	> 12 months	≥ 3 months		P	P	> 3 months	> 2 years	NS
	Adults	A	(less common)	A	A	A	NS	A		A	A		A	A	NS
*S. agalactiae*	Neonates		N			N	N	N	N		N	N	≤ 7 days		NS
	Paediatric				< 3 months						< 24 months	< 3 months			NS
	Adults			A	(rare)					A					NS
*H. influenzae*	Neonates														NS
	Paediatric	P	(unvaccinated)				NS		≥ 3 months		< 24 months		3 months to 5 years		NS
	Adults	(rare)	(rare)	A	(rare)	A	NS	A		A					NS
*L. monocytogenes*	Neonates		(rare)			N	N		N		N	N	> 7 days		NS
	Paediatric	P											≤ 3 months		NS
	Adults	A	> 50 years	A	(rare)	A		A		> 60 years	> 50 years			> 50 years	NS
*E. coli*	Neonates		N				N	N	N		N	N	≤ 7 days		NS
	Paediatric	P			< 3 months (rare)						< 24 months				NS
	Adults	A		A							> 50^#^				NS
*Klebsiella* spp.	Neonates										N		≤ 7 days		NS
	Paediatric	P													
	Adults	A								A^^^					
*Enterobacter* spp.	Neonates														
	Paediatric	P													
	Adults	A				A				A					
*Pseudomonas aeruginosa*	Neonates												≤ 7 days		
	Paediatric	P													
	Adults	A		A		A^^^				A					
*Proteus mirabilis*	Neonates														
	Paediatric	P													
	Adults	A													
*Salmonella* spp.	Neonates												≤ 7 days		
	Paediatric														
	Adults														
*S. aureus*	Neonates						(very rare)								
	Paediatric	P													
	Adults	A	A (rare)	A		A									

*Abbreviations: N* neonates, *P* paediatric populations, *A* adults, *NS* non-specified population, *EFNS* European Federation of Neurological Societies, *ESCMID* European Society of Clinical Microbiology and Infectious Diseases, *DSI* Dansk Selskab for Infektionsmedicin, *SPILF* Société de Pathologie Infectieuse de Langue Française, *DGN: BM* Deutsche Gesellschaft fur Neurologie: Bakterielle Meningoenzephalitis, *HPSC* Health Protection Surveillance Centre, *NVN* Nederlandse Vereniging voor Neurologie, *NICE* The National Institute for Health and Care Excellence, *UKJSS* UK Joint Specialist Societies, *IDSA* Infectious Diseases Society of America, *AEPED* Asociación Española de Pediatría, *MSF* Médicins Sans Frontiéres, *NNF* Norsk Nevrologisk Forening, *PHE: ME* Public Health England: Meningoencephalitis

*Focused on invasive meningococcal disease only

^^^And other Gram-negative

^#^Gram-negative bacteria in A > 50 years

^^^^Additional recommendations: *B. burgdorferi, M. pneumoniae*, *Chlamydia trachomatis, M. tuberculosis.* Two CMGs focused on *N. meningitides* only [20, 21] and are therefore not presented in the table

The different epidemiology for neonatal meningitis was reflected in the eight CMGs providing recommendations for neonates. All recommended testing for GBS, most also for *E. coli* (*n* = 7) and *L. monocytogenes* (*n* = 7). There were variations in the definitions of risk groups for neonates and infants. The CMG produced by MSF recommended testing for GBS and Gram-negative bacteria up to 7 days of age and *S. pneumoniae* and *L. monocytogenes* in more than 7-day-old neonates [38]. Two CMGs recommended using the neonate recommendations in infants up to 3 months of age [24, 38]. For infants beyond the neonatal period, most recommended testing for *N. meningitidis* (*n* = 11), *S. pneumoniae* (*n* = 10) and Hib (*n* = 6), again with variations in the age cut-offs.

### Empirical treatment

#### Viral encephalitis/meningoencephalitis

Although a wide range of viruses can cause CNS infections, the treatment options are limited. Early treatment using acyclovir (I.V.) pending diagnosis was recommended by the CMGs focused on viral CNS infections that included treatment recommendations [7, 22, 26–33] since early treatment with acyclovir has been associated with a lower risk of sequelae and death from the most commonly diagnosed cause, HSV [7] (Table 6). The recommended dose for adults or unspecified populations was 10 mg/kg I.V. with a recommended duration varying from 10 to more than 14 days. A CMG from France (2017) recommended the addition of amoxicillin in adults to cover for risk of *L. monocytogenes* [26]. The dose for children ranged from 10 to 20 mg/kg acyclovir I.V. for 14 to more than 21 days [27, 28], to 20 mg/kg I.V. for neonates for at least 14 days [27, 28, 32]. It was noted that treatment and duration should be assessed depending on additional symptoms and modified depending on diagnostic results.

**Table 6:**
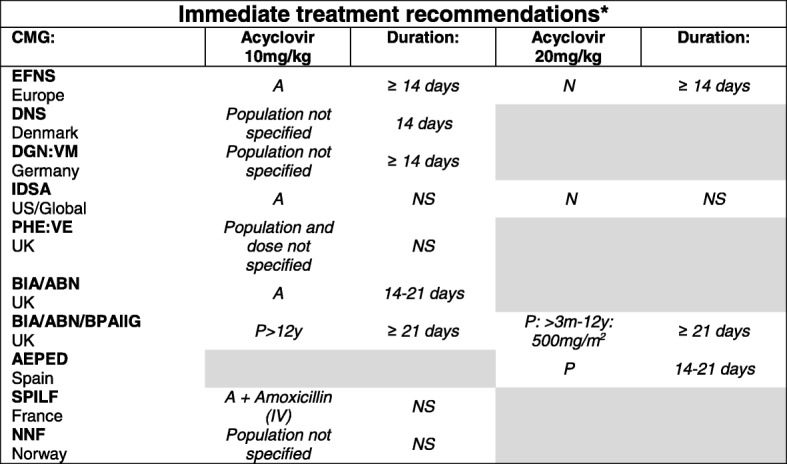
Empirical treatment recommendations for suspected viral encephalitis and meningoencephalitis

The table shows the CMGs that included recommendations for empirical treatment

*Abbreviations*: *N* neonates, *P* paediatric populations, *A* adults, *NS* non-specified population, *EFNS* European Federation of Neurological Societies, *DNS* Dansk Neurologisk Selskab, *DGN: VM* Deutsche Gesellschaft für Neurologie: Virale Meningoeczephalitis, *PHE: VE* Public Health England:Viral Encephalitis, *BIA: ABN*: British Infection Association: Association of British Neurologists, *BPAIIG* The British Paediatric Allergy Immunology and Infections Group, *AEPED* Asociación Española de Pediatría, *SPILF* Société de Pathologie Infectieuse de Langue Française, *PHE: ME* Public Health England: Meningoencephalitis, *NNF* Norsk Nevrologisk Forening

*Initial empirical treatment recommendations should be reviewed pending diagnostic results

#### Bacterial meningitis

All 15 CMGs covering treatment recommended urgent administration of antibiotics on clinical suspicion of bacterial meningitis (Table 7). Thirty-three percent (*n* = 5/15) specified a time-frame for administration, ranging from within one [2, 5] to three [34, 36, 37] hours. Forty-seven percent (*n* = 7/15) recommended pre-hospital antibiotics if the patient initially presents to a healthcare setting outside of a hospital. One CMG noted that there are no prospective clinical data on the relationship of the timing of antimicrobial administration to clinical outcome in patients with bacterial meningitis, but since it is a neurologic emergency, appropriate therapy is recommended as soon as possible after the diagnosis is considered likely [44]. It was highlighted that the choice of antibiotics should be informed by risk factors for different aetiologies, such as age and risk of reduced susceptibility to penicillin and third-generation cephalosporins [5]. This was reflected in the CMGs, though with variations in specification of at-risk groups. Most recommended a third-generation cephalosporin alone [5, 34–36, 38, 39, 44] or in combination with a penicillin for all adults [2, 23, 37], “elderly” [36] and over 50 [5, 39, 44] or over 60 years [2], due to higher risk of *L. monocytogenes* infection in these risk groups [5, 34, 36, 39]. Most guidelines that provided recommendations for neonates recommended a third-generation cephalosporin plus penicillin [5, 20, 23, 24, 35, 38, 40, 44] or alternatively an aminoglycoside with penicillin [5, 35, 38, 44]. However, the age cut-off for these recommendations varied, ranging from 1 [5, 23, 44], to 2 [35], or 3 months of age [20, 24, 38, 40]. For older infants and children, there was consensus on the recommendations of a third-generation cephalosporin alone [5, 19–21, 23, 34–36, 38]. The guideline by MSF recommended a cephalosporin in an epidemic context where *N. meningitides* is the most likely pathogen, and addition of cloxacillin if associated skin or umbilical cord infection for all age-groups [38].

**Table 7:** Empirical treatment recommendations for suspected bacterial meningitis

Initial treatment recommendations*					
CMG	3rd-generation cephalosporin (ceftriaxone^^^ or cefotaxime)	3rd generation-cephalosporin (ceftriaxone^^^ or cefotaxime) plus a penicillin (amoxicillin, ampicillin or penicillin)	Aminoglycoside (gentamicin) plus a penicillin (amoxicillin or ampicillin)	Add: glycopeptide (vancomycin)	Add: corticosteroids (before or with first dose of antibiotics)
EFNS Europe	P, A	E		Older children and adults**	Yes
ESCMID Europe	P, A	N, A > 50 years, or if risk factor for *L. monocytogenes*	N	**,^^^	Yes^^^ up to 4 h post-antibiotics
DSI Denmark	A	A if risk of *L. monocytogenes*			Yes
SPILF France	P, A	P, A if suspected L. monocytogenes°		If S. pneumoniae	Yes^″^
DGN: BM Germany	A	A		A**	Yes
HPSC Ireland	P > 2 m, A	N, P < 2 months	N, P < 2 months	**,^^^	Yes up to 24 h post-antibiotics
NVN Netherlands	P, A	N, A			Yes
MHSSE^#^ Spain	P				Yes
NICE UK	P > 3 m	N, P < 3 months		If travel outside of the UK	Yes^^^^ up to 12 h post-antibiotics
UKJSS UK	A	A > 60 years		Pending travel history	Yes up to 12 h post-antibiotics
SIGN^#^ Scotland	P > 3 m	N, P ≤ 3 months			Yes up to 24 h post-antibiotics
IDSA USA/Global	P, A	N, A > 50 years	N	P, A	P^^^, infants if Hib, A
AEPED Spain	P	N, P ≤ 3 months		**,^^^	Yes
MSF Global	P > 3 m, A	N, P ≤ 3 months	N, P ≤ 3 months		Yes^^^
NNF Norway	NS	NS			Yes

The table shows empirical treatment recommendations for different risk groups

*Initial recommendations to be reviewed as appropriate pending diagnostic results

**If suspicion of reduced sensitivity to penicillin

^^^Not to neonates

^^^^Not to infants < 3 months

^″^Not to immunosuppressed

^#^Focused on *N. meningitidis* only

*Abbreviations*: *N* neonate, *P* paediatric populations, *A* adults, *E* elderly, *NS* non-specified population*, EFNS* European Federation of Neurological Societies, *ESCMID* European Society of Clinical Microbiology and Infectious Diseases, *DSI* Dansk Selskab for Infektionsmedicin, *SPILF* Société de Pathologie Infectieuse de Langue Française, *DGN* Deutsche Gesellschaft für Neurologie, *BM* Bakterielle Meningoenzephalitis, *HPSC* Health Protection Surveillance Centre, *NVN* Nederlandse Vereniging voor Neurologie, *MHSSE* Ministry of Health, Social Services and Equality, *NICE* The National Institute for Health and Care Excellence, *UKJSS* UK Joint Specialist Societies, *SIGN* Scottish Intercollegiate Guidelines Network, *IDSA* Infectious Diseases Society of America, *AEPED* Asociación Española de Pediatría, *MSF* Médecins Sans Frontières, *NNF* Norsk Nevrologisk Forening

Many CMGs recommended the addition of vancomycin [2, 5, 34–37, 39, 40] alternatively rifampicin [2, 5, 37, 39] if there is suspicion of reduced sensitivity to penicillin, except for neonates. However, the CMGs produced by IDSA (USA) and NICE (UK) gave different advice. The first recommended vancomycin to everyone except neonates (< 28 days old) [44], whereas the CMG by NICE recommended vancomycin to all returning travellers or those with recent prolonged or multiple exposure to antibiotics within the past 3 months, to cover risk of penicillin-resistant strains of *S. pneumoniae* [24].

All CMGs focused on bacterial CNS infections recommended adjunctive corticosteroids therapy before or with first dose of antibiotics. Some recommended it up to a few hours [37], 4 h [5, 39], 12 h [2, 24] or 24 h post-antibiotics [20, 35]. Four explicitly did not recommend steroids for patients with immunosuppression [34] or for neonates [5, 24, 44]. One CMG noted that corticosteroids in infants is controversial and only recommended for Hib meningitis [44]. It was highlighted that corticosteroids can reduce inflammation and brain oedema and has in some studies shown benefits of reducing rates of complications and improving outcomes in patients with meningitis, but also that some studies have raised concerns about potential side effects [2].

## Discussion

The data highlights the wide range of CMGs for acute, community-acquired CNS infections in use across Europe and variations in quality, clinical case definitions for guiding identification and in initial clinical recommendations. Most CMGs were produced by national or European organisations. Several survey respondents reported using CMGs produced by other countries in or outside of Europe, which may have implications for timely identification of causative pathogens and use of antibiotics, unless they are adapted to regional epidemiology.

There were several high-quality CMGs that adhered to most of the standards set out in the AGREE II tool. All of the highest scoring CMGs were focused on bacterial aetiologies. Many were more than 3 years old, which is the recommended time-frame for re-assessment of validity [18, 45, 46]. This is a concern in light of the rapidly changing epidemiology of infections and antimicrobial resistance. The most recent Europe-wide CMG on bacterial meningitis was published in 2016 [5], whilst the most recent Europe-wide CMG for viral CNS infections was updated in 2010 [32]. Though there might be additional CMGs not identified through the review or survey, this, together with the fact that many survey respondents used international CMGs, highlights a need for an updated European-wide CMG covering viral CNS infections in adult and paediatric populations that can be adapted nationally.

There was a general consensus on diagnostic methods, but wider variations in infectious disease differential diagnostics recommendations, especially for paediatric and elderly populations. There was also general consensus on the most common bacterial causative agents for adults and older children, but variations in differential diagnostic recommendations for infants, neonates and elderly and in the definitions of these risk groups. This is illustrated by the different risk groups identified for *L. monocytogenes* infection, which in two CMGs were defined as adults over 50 years [5, 44], whereas over 60 years in another [2]. It is also illustrated by the wide variations in the definition of risk groups for GBS, defined as younger than 1 week [38], 28 days [2, 5, 23, 24, 45], 3 months [34, 40] or 24 months [44] in the recommendations for neonates and infants.

In regard to empirical treatment, there was general consensus on recommended initial therapy for suspected viral aetiologies, pending diagnostics. Moreover, there was a consensus across CMGs on the need for urgent I.V. antibiotics on clinical suspicion of bacterial meningitis. However, only about half recommended pre-hospital antibiotics when presenting outside of a hospital setting. All CMGs recommended a third-generation cephalosporin for suspected bacterial aetiologies, to cover the most common pathogens in adults and children, but with variations in recommendations for adding penicillin to cover *L. monocytogenes*. Most of the European CMGs recommended the addition of vancomycin or rifampicin if decreased susceptibility to penicillin or third-generation cephalosporin is suspected based on geographical regions visited, whereas the CMG by NICE recommended it to all returning travellers [24]. The CMG by IDSA aimed at all global settings recommended vancomycin to everyone beyond neonatal age [44], which highlights the risk of inappropriate antibiotics usage unless adapted to regional epidemiology.

Some of the variations in recommendations can be explained by regional differences in epidemiology and risks of reduced sensitivity to antimicrobial agents. ECDC data from 2015 shows wide variations in reduced penicillin resistance rates of *S. pneumoniae* from 0.6% in Belgium, to more than 20% in Slovakia, Bulgaria, France, Spain, Iceland, Poland, Malta and Romania [47]. The variations in recommendations seen between CMGs produced in Europe, compared to those produced outside of Europe, but used by clinicians in Europe, indicate risks of inappropriate differential diagnostic requests and antimicrobial treatment regimes, unless the guidelines are adapted to national settings. This may lead to delayed identification of diseases and inappropriate initial management. The variations seen in recommendations for paediatric and elderly populations and the limited number of CMGs covering these populations also indicate a need to ensure equity in access to CMGs covering all different at-risk population, as well as region-appropriate recommendations.

The review highlights a need for clinicians to ensure that CMGs are robustly developed, up-to-date and appropriate for the setting and population. In an increasingly global world, it is important to ensure CMGs also address risks of travel-imported infections. Some CMGs addressed this by providing tables based on risk factors [7, 10], or links to websites with up-to-date international surveillance data and risk assessments [5], such as the ECDC and the World Health Organizations websites. This highlights the importance of these websites to provide current data about the epidemiology of circulating infections and the risk of antibiotic-resistant strains globally. Other variations reflect limitations in the evidence available, such as the definition of risk groups for infections and effective treatment strategies for these. Furthermore, the limitations in evidence was also reflected in the variations in recommended timing of antibiotics and corticosteroids for bacterial CNS infections, which was also noted in a recent review [3].

This review highlights important differences in quality, coverage and initial clinical recommendations between the CMGs. The AGREE II tool was useful for assessing the quality of the CMGs. It may be used together with other tools, such as the 4-item Global Rating Scale (GRS), but a comparison showed that the GRS is less sensitive in detecting differences in guideline quality [48]. There are limitations to the review, in only including CMGs which were published or accessible via the survey, and despite the lack of language restrictions, most CMGs identified were produced in English or by EU/EEA member states. Furthermore, the data extraction was limited to parameters associated with the early identification and initial management of syndromes of community-acquired CNS infections, to assess variations which could impact on the early identification, management and control of emerging infections with epidemic potential. Antibiotic dosage was not presented, as this is a clinical decision which may be affected by individual patient characteristics. The review does not attempt to create a new guideline, but to highlight important limitations and differences in CMGs in use across Europe, identifying the need to update recommendations and harmonise standards in order to inform future research needs and CMG development.

Despite these limitations, this review highlights variations in quality and recommendations between the CMGs, which may be barriers for the rapid identification and management of CNS infections which may have an impact on health outcomes and timely identification of emerging outbreaks. Considering the resources required to develop complex, evidence-based CMGs, not all health systems might have the resources required. A “framework-CMG” produced by an international network of appropriate experts and stakeholders can provide a useful model for CMG development, as evident by the high-quality CMGs identified, some of which have been adopted by clinicians in several countries. This internationally produced framework CMG would need to address regional risks and consider resources for regular review, updating and dissemination. This model can also improve harmonisation of case definitions and recommendations, which can facilitate equity in access to best available evidence-based recommendations, rapid identification of emerging infections and clinical, research and public health responses to epidemics. The recent guideline on bacterial CNS infections produced by ESCMID [5] is a good example of a robust, comprehensive CMG aimed at a Europe-wide audience, which can serve as a model to be adapted to regional epidemiology as appropriate and monitored for uptake across the region.

## Conclusions

This review highlights variations in the quality and recommendations of CMGs for community-acquired CNS infections in use across Europe. A harmonised European framework-CMG with adaptation to local epidemiology and risks may improve access to up-to-date CMGs and the early identification and management of (re-) emerging CNS infections with epidemic potential. The review particularly highlights the need for an updated European CMG for infectious encephalitis, which covers all risk groups, including paediatric and elderly populations. Further research into risk groups for infections and effective treatment strategies to target these populations is required.

## Additional files

Additional file 1: AGREE II Instrument for the Quality Assessment of Clinical Management Guidelines (PDF 76 kb)

Additional file 2: Clinical management guidelines included in the review (PDF 57 kb)

## Data Availability

All data generated or analysed during this study are included in this published article [and its Additional files].

## Abbreviations

AEPED: Asociación Española de Pediatría
AGREE II: Appraisal of Guidelines for Research and Evaluation II
ALOC: Altered level of consciousness
AMS: Altered mental status
BIA: ABN: British Infection Association: Association of British Neurologists
BPAIIG: The British Paediatric Allergy Immunology and Infections Group
CLIN-Net: Clinical investigator network
CMG: Clinical management guideline
CMV: Cytomegalovirus
CNS: Central nervous system
COMBACTE-Net: Combatting antibiotic resistance in Europe-network
CSF: Cerebral spinal fluid
CT: Computerised tomography
DGN: BM: Deutsche Gesellschaft für Neurologie: Bakterielle Meningoenzephalitis
DGN: VM: Deutsche Gesellschaft für Neurologie: Virale Meningoeczephalitis
DNS: Dansk Neurologisk Selskab
DSI: Dansk Selskab for Infektionsmedicin
EBV: *Epstein-Barr virus*
ECDC: European Centre for Disease Prevention and Control
EEA: European Economic Area
EEG: Electroencephalogram
EFNS: European Federation of Neurological Societies
ESCMID: European Society of Clinical Microbiology and Infectious Diseases
EU: European Union
GBS: *Streptococcus agalactiae*; group B streptococcus
GRS: Global Rating Scale
HHV: Human herpesvirus
Hib: *Haemophilus influenzae* type B
HIV: Human immunodeficiency virus
HPSC: Health Protection Surveillance Centre
HSV: Herpes simplex virus
ICP: Intracranial pressure
IDSA: Infectious Diseases Society of America
IEC: International Encephalitis Consortium
I.V.: Intravenous
JEV: Japanese encephalitis virus
LP: Lumbar puncture
MeSH: Medical subject headings
MHSSE: Ministry of Health, Social Services and quality
MRI: Magnetic resonance imaging
MSF: Médicins Sans Frontières
NICE: The National Institute for Health and Care Excellence
NNF: Norsk Nevrologisk Forening
NVN: Nederlandse Vereniging voor Neurologie
PCR: Polymerase chain reaction
PHE: ME: Public Health England: Meningoencephalitis
PHE: VE: Public Health England: Viral Encephalitis
PREPARE: Platform for European Preparedness Against (Re-)emerging Epidemics
PROSPERO: International Prospective Register of Systematic Reviews
RSV: Respiratory syncytial virus
SIGN: Scottish Intercollegiate Guidelines Network
SPILF: Société de Pathologie Infectieuse de Langue Française
TBEV: Tick-borne encephalitis virus
TRIP: Turning research into practice
UK: United Kingdom
UKJSS: UK Joint Specialist Societies
VZV: *Varicella-zoster virus*
WNV: West Nile virus
