# Supplementary material for: A systematic review of clinical guidelines on the management of acute, community-acquired CNS infections
Source: BMC Med. 2019 Sep 6;17:170. doi: 10.1186/s12916-019-1387-5 (PMC6729038; doi:10.1186/s12916-019-1387-5)
Supplement: Supplementary file 1 — AGREE II Instrument for the Quality Assessment of Clinical Management Guidelines (PDF 76 kb) [file 12916_2019_1387_MOESM1_ESM.pdf]

Additional file 1. AGREE II Instrument for the Quality Assessment of Clinical Management Guidelines

AGREE II Domain	AGREE II Item
Scope and Purpose	The overall objective(s) of the guideline is (are) specifically described
	The health question(s) covered by the guideline is (are) specifically described
	The population (patients, public, etc.) to whom the guideline is meant to apply is specifically described.
Stakeholder Involvement	The guideline development group includes individuals from all relevant professional groups
	The views and preferences of the target population (patients, public, etc.) have been sought
	The target users of the guideline are clearly defined
Rigour of Development	Systematic methods were used to search for evidence.
	The criteria for selecting the evidence are clearly described
	The strengths and limitations of the body of evidence are clearly described
	The methods for formulating the recommendations are clearly described
	The health benefits, side effects, and risks have been considered in formulating the recommendations
	There is an explicit link between the recommendations and the supporting evidence.
	The guideline has been externally reviewed by experts prior to its publication
	A procedure for updating the guideline is provided
Clarity and Presentation	The recommendations are specific and unambiguous
	The different options for management of the condition or health issue are clearly presented
	Key recommendations are easily identifiable
Applicability	The guideline describes facilitators and barriers to its application
	The guideline provides advice and/or tools on how the recommendations can be put into practice
	The potential resource implications of applying the recommendations have been considered
	The guideline presents monitoring and/or auditing criteria
Editorial Independence	The views of the funding body have not influenced the content of the guideline
	Competing interests of guideline development group members have been recorded and addressed
**Overall Score**	
Overall Quality	1 – 7 (1=lowest possible quality, 7=highest possible quality)
Recommended for Use	Yes/Yes, with modifications/No
