# Supplementary material for: A systematic review of clinical guidelines on the management of acute, community-acquired CNS infections
Source: BMC Med. 2019 Sep 6;17:170. doi: 10.1186/s12916-019-1387-5 (PMC6729038; doi:10.1186/s12916-019-1387-5)
Supplement: Supplementary file 2 — Clinical management guidelines included in the review (PDF 57 kb) [file 12916_2019_1387_MOESM2_ESM.pdf]

Guidelines	Year	Target region	Authorising Body	Focus	Target Population	Language
**Case definitions, diagnostic algorithms, and priorities in Encephalitis: Consensus statement of the International Encephalitis Consortium (IEC)**	2013	US/Global	International Encephalitis Consortium (IEC)	Viral encephalitis	A, P	English
**Viral meningoencephalitis: a review of diagnostic methods and guidelines for management (EFNS)**	2010	Europe	European Federation of Neurological Societies (EFNS)	Viral meningoencephalitis	U	English
**Akut infektiøs encefalitis(DNS)**	2017	Denmark	Dansk Neurologisk Selskab (DNS)	Viral encephalitis	U	Danish
**Virale Meningoenzephalitis (DGN)**	2018	Germany	Deutsche Gesellschaft für Neurologie (DGN)	Viral meningoencephalitis	U	German
**The Management of Encephalitis: Clinical Practice Guidelines by the Infectious Diseases Society of America (IDSA)**	2008	US/Global	Infectious Diseases Society of America (IDSA)	Viral encephalitis	A,P	English
**UK Standards for Microbiology Investigations: Investigation of Viral Encephalitis (PHE:VE)**	2014	UK	Public Health England (PHE)	Viral encephalitis	U	English
**Management of suspected viral encephalitis in adults (BIA/ABN)**	2012	UK	British Infection Association (BIA), Association of British Neurologists (ABN)	Viral encephalitis	A	English
**Management of suspected viral encephalitis in children (BIA/ABN/BPAIIG)**	2012	UK	BIA, ABN, British Paediatric Allergy Immunity and Infection Group (BPAIIG)	Viral encephalitis	P (<16 yo)	English
**Encefalitis (AEPED)**	2011	Spain	Asociación Española de Pediatria (AEPED)	Viral encephalitis	P	Spanish
**Guidelines on the management of infectious encephalitis in adults (SPILF)**	2017	France	Société de Pathologie Infectieuse de Langue Française (SPILF)	Infectious encephalitis	A	English (French)
**Veileder I Akuttnevrologi: Infeksjoner/NevroNEL (NNF)**	2017	Norway	Norsk Nevrologisk Forening (NNF)	Infectious encephalitis	U	Norwegian
**UK Standards for Microbiology Investigations: Meningoencephalitis (PHE:ME)**	2014	United Kingdom	Public Health England (PHE)	Infectious meningoencephalitis	U	English
**EFNS guideline on the management of community-acquired bacterial meningitis: report on an EFNS Task Force on acute bacterial meningitis in older children and adults (EFNS)**	2008 (rev. 2010)	Europe	European Federation of Neurological Societies (EFNS)	Bacterial meningitis	A, P (older children)	English
**Diagnosis and treatment of acute bacterial meningitis (ESCMID)**	2016	Europe	European Society of Clinical Microbiology and Infectious Diseases (ESCMID)	Bacterial meningitis	A, P	English
**Rekommandationer for initial behandling af akut bakteriel meningitis hos voksne (DSI)**	2018	Denmark	Dansk Selskab for Infektionsmedicin (DSI)	Bacterial meningitis	A	Danish
**Practice guidelines for acute bacterial meningitidis (except newborn and nosocomial meningitis)(SPILF)**	2008	France	Société de Pathologie Infectieuse de Langue Française (SPILF)	Bacterial meningitis	A, P	English (French)
**Ambulant erworbene bakterielle (eitrige) Meningoenzephalitis im Erwachsenalter (DGN:BM)**	2018	Germany	Deutsche Gesellschaft für Neurologie (DGN)	Bacterial meningitis	A	German
**Guidelines for the early clinical and public health management of bacterial meningitis (including Meningococcal Disease) (HPSC)**	2016	Ireland	Health Protection Surveillance Centre (HPSC)	Bacterial meningitis	A, P	English
**Bacteriële Meningitis (NVN)**	2013	Netherlands	Nederlandse Vereniging voor Neurologie (NVN)	Bacterial meningitis	A, P	Dutch
**Clinical Practice Guideline on the Management of Invasive Meningococcal Disease (MHSSE)**	2013	Spain	Ministry of Health, Social Services and Equality (MHSSE)	Meningococcal Disease	P	English, Spanish
